# Relevance of Activity Tracking With Mobile Devices in the Relationship Between Physical Activity Levels and Satisfaction With Physical Fitness in Older Adults: Representative Survey

**DOI:** 10.2196/12303

**Published:** 2019-03-06

**Authors:** Anna Schlomann, Alexander Seifert, Christian Rietz

**Affiliations:** 1 Department of Special Education and Rehabilitation Science University of Cologne Cologne Germany; 2 “Dynamics of Healthy Aging” University Research Priority Program University of Zurich Zurich Switzerland; 3 Center of Competence for Gerontology University of Zurich Zurich Switzerland; 4 Mixed Methods Research Heidelberg University of Education Heidelberg Germany

**Keywords:** physical fitness, wearable electronic devices, smartphone, mobile phone, aged, satisfaction, fitness trackers

## Abstract

**Background:**

Physical activity has been shown to positively affect many aspects of life, and the positive relationship between physical activity levels and health is well established. Recently, research on the interrelationship between physical activity levels and subjective experiences has gained attention. However, the underlying mechanisms that link physical activity levels with subjective experiences of physical fitness have not been sufficiently explained.

**Objective:**

This study aimed to explore the role of physical activity tracking (PAT) in the relationship between physical activity levels and satisfaction with physical fitness in older adults. It is hypothesized that higher levels of physical activity are associated with a higher satisfaction with physical fitness in older adults and that this positive association is stronger for older people who use mobile devices for PAT.

**Methods:**

As part of this study, 1013 participants aged 50 years or older and living in Switzerland were interviewed via computer-assisted telephone interviews. Bivariate and multivariate analyses were applied. The interaction effects between physical activity levels and PAT were evaluated using multiple linear regression analysis.

**Results:**

Descriptive analyses showed that 719 participants used at least 1 mobile device and that 136 out of 719 mobile device users (18.9%) used mobile devices for PAT. In the multivariate regression analysis, frequent physical activity was found to have a positive effect on satisfaction with physical fitness (beta=.24, *P*<.001). A significant interaction effect between physical activity levels and PAT (beta=.30, *P*=.03) provides some first evidence that the positive effects of physical activity on satisfaction with physical fitness can be enhanced by PAT.

**Conclusions:**

The results indicate the potential of PAT to enhance the physical fitness of older adults. However, the results also raise new issues in this context. Recommendations for further research and practice include the acquisition of longitudinal data, a more detailed observation of durations of use, and the development of devices for PAT considering health psychology and gerontology theories.

## Introduction

### Background

Physical inactivity is a major health risk facing people worldwide, especially older adults [1]. Many people are not adequately physically active, and physical activity level decreases with age [2]. Various organizations, including the World Health Organization [3] and the US Department of Health and Human Services [4], have discussed the negative effects of insufficient physical activity at the public health policy level. Both these organizations have developed guidelines for the appropriate physical activity levels of each age group: for adults, at least 150 min a week of moderate-intensity or 75 min of vigorous-intensity physical activity is recommended [4]. The positive relationship between physical activity levels and health across all age groups is well established and widely documented [5,6]. An appropriate level of physical activity can also contribute to healthier aging processes [7] and prevent age-related cognitive decline [8]. Furthermore, it was demonstrated that physical activity not only positively affected health but also feelings of well-being such as happiness [9]. Self-reported fitness has also been shown to be a good predictor of mortality [10]. However, research also shows that general guidelines for physical activity do not appeal to everyone: women, for example, were more motivated by the 10,000 steps message than men [11], and general recommendations for physical activity levels that are not adapted to individual capabilities might even lead to feelings of overexertion in older adults [12].

Against this background, it is important not only to look at absolute levels of physical activity but also to consider their subjective evaluation. This includes the question of how satisfied an individual is with his or her physical activity levels and how this satisfaction is achieved. Recommendations for physical activity levels only provide a general framework. A good measure of individual satisfaction is not a global goal of, for example, 10,000 steps or 150 min, rather, it is the subjective estimation of the positive effects of physical activity on health and well-being.

### Effects of Physical Activity on Subjective Experiences

Research on the interrelationship between physical activity levels and subjective experiences has gained attention in recent years. Empirical research has shown the positive effects of physical activity on health-related quality of life [13], subjective well-being, and life satisfaction [14-18]. Further research showed that walking and other types of physical activity significantly contributed to individual happiness in ordinary least squares (OLS) regression analyses [9]. However, the underlying mechanisms that link subjective satisfaction with physical fitness and actual physical activity levels have not yet been sufficiently explored in empirical research [19]. Ehlers et al [20] summarized current findings on the effects of physical activity on well-being in older adults, and the positive effects of physical activity on both negative and positive psychological states have been demonstrated. Nevertheless, the authors conclude that further research is needed to explain the specific effects of dose, mode, and underlying mechanisms on the positive effects of physical activity [20].

There has been some work on the age-specific effects of physical activity on subjective well-being. Pawlowski et al [17] showed that, although the effects are generally low, the positive effects of physical activity on subjective well-being increase with age. In another study, well-being was positively related to physical activity and physical function in older adults [15]. Qualitative research showed that older adults perceive physical activity as a behavior related to health and well-being [21]. Building on these findings, we focus in more detail on satisfaction with physical fitness levels as one example of subjective well-being and its relationship with physical activity levels.

### Relevance of Mobile Physical Activity Tracking for the Effects of Physical Activity

Mobile tracking technologies such as activity trackers and other wristband sensors that track physical activity (eg, activity monitors, activity wristbands, and smartwatches), as well as apps on smartphones and tablets (eg, ActivityTracker, Runkeeper, and MyFitnessPal), are growing in popularity [22-24] and might be of relevance in this context. It has been shown that an individual’s level of physical activity can actually increase through the use of physical activity tracking (PAT) with mobile devices [25,26]. This has also been shown for the group of older people in a small experimental study [27]. The apps make use of gamification elements such as badges or rank lists that facilitate goal setting and increase self-efficacy [28-30]. Furthermore, the use of mobile devices for PAT can help users and health care professionals understand users’ health and symptoms, owing to the possibility of drawing correlations between user behavior and health outcomes [31]. As described by Morgan [32], “many of these technologies allow individuals to self-track, make records of and respond to a range of previously invisible biomedical and behavioral data.” In this sense, using mobile technologies for PAT increases access to relevant health-related data [32].

Despite a growing number of studies on PAT, the relevance of PAT in terms of the relationship between physical activity levels and subjective experiences of satisfaction with physical fitness has not been studied in detail yet. In addition, research on PAT has traditionally focused on young or middle-aged individuals [33-35] or individuals who are already physically active [36]. However, health-related issues and disease management gain importance as individuals age [37]. Qualitative research provided indications that PAT generally influences feelings of well-being, emotions, and awareness for physical activity [38]. Furthermore, a scoping review showed that older adults are generally interested in the use of technologies for health purposes and disease prevention [39]. In a randomized controlled trial, it was shown that an internet-based physical activity intervention that included accelerometry improved older adults’ quality of life [40], which is a further indicator of the relevance of PAT in this context.

### Research Questions and Hypotheses

The objective of this study was to contribute to the literature on the positive effects of physical activity levels by examining the importance of PAT in this context. More precisely, we are interested in whether the relationship between physical activity levels and older adults’ satisfaction with physical fitness is influenced by PAT with mobile devices (ie, activity trackers, smartwatches, smartphones, and tablets). Given the empirical evidence on the interrelation between physical activity levels and satisfaction with physical fitness in general [9,17,20], we generally assume a positive relationship between physical activity levels and satisfaction with physical fitness. Therefore, our first hypothesis (H1) is as follows:

Higher levels of physical activity are associated with a higher satisfaction with physical fitness.

Mobile technologies for PAT allow for the quantification of the levels of physical activity, enable goal setting, and make achievements more visible [22,23]. Therefore, their use adds to the positive effect of physical activity levels on satisfaction with physical fitness. Our second hypothesis (H2) is as follows:

The positive association between physical activity levels and satisfaction with physical fitness is stronger for people who use mobile devices for PAT.

## Methods

### Sample and Data

This secondary analysis is based on a survey performed in Switzerland [41]. In November 2016, 1013 adults, aged 50 years and older, were interviewed from the German- and French-speaking regions of Switzerland (representing approximately 92% of the entire Swiss population in that age group) using computer-assisted telephone interview. The response rate of the survey was 17.71% (1013/5719). Participation in the telephone interview was voluntary, and participants were asked for approval at the beginning of the interview. We consulted the Ethics Committee of the Faculty of Arts and Social Sciences of the University of Zurich to assess the ethics requirements for the study. The authors were required to complete a checklist to self-assess the ethical safety during the study [42]. On the basis of the outcomes of this self-assessment, no further application for approval to the ethics committee was necessary. In the first publication of study results, the authors [41] described the use of mobile devices among older adults in Switzerland and analyzed the ownership of smartphones, tablets, smartwatches, and physical activity trackers in more detail. The authors found that men, younger individuals, and people with a strong interest in new technology had a higher likelihood of using mobile devices. The secondary analysis in this paper focuses on the relationship of use with psychological variables.

A standardized questionnaire with 24 questions about users’ sociodemographic information and mobile device use for PAT was administered. A random sample of the permanent resident population of Switzerland aged 50 years and older was chosen from the commercial AZ-Direct database (based on the public phone book). No restrictions were imposed on upper age, current mobile device use, or type of housing. This study included a representative sample of all age groups examined across gender, education, and language region. Within the whole sample, 70.98% (719/1013) used at least 1 mobile device—such as a smartphone, tablet, and/or smartwatch—in their everyday life, whereas 29.02% (294/1013) reported that they did not use any of these devices. For the purpose of this paper, only the sample group of individuals who used any of the aforementioned mobile devices (n=719) was considered in the analyses. Participants’ age in this group ranged from 50 to 88 years, with a mean age of 62.7 years (SD 9.45); 50.5% (363/719) were female; and 49.5% (356/719) were male.

### Measures

Mobile PAT comprises activity trackers (ie, wristbands with accelerometer technology for monitoring and tracking fitness-related behavior, mostly based on counting steps and time periods of physical activity), smartwatches (ie, computerized wristbands with various functionalities and apps similar to those of smartphones, which run on their own operating systems), and smartphone or tablet apps; all of these can be used for tracking physical activity. We generally asked whether the respondents used apps or devices for the purpose of tracking physical activity; however, we do not have information regarding whether the respondents used the devices or apps actively or passively (eg, active measurement or automatic recording of steps). The use of these devices and apps was measured by self-report (1=never, 2=seldom, 3=once a week, and 4=daily). Individuals who used any of these devices or apps for PAT at least once a week are referred to hereafter as the *physical activity tracking group* (PAT group). Individuals who did not track their physical activity with any of these devices or apps or reported to use them less than once a week are referred to as the *no physical activity tracking group* (noPAT group). This formation of groups allows us to focus the analyses on regular users of PAT. All individuals belonging to the noPAT group do use at least 1 mobile device (ie, smartphone, tablet, and/or smartwatch) for purposes other than tracking their physical activity. This computed group variable was used as a dummy variable in the analyses (PAT group=1, noPAT group=0). Individuals who did not use any mobile device (n=294) were not considered in the analyses. This was done both to focus the analyses on the relationship of using PAT with satisfaction with physical activity and to include only those people with a comparable use behavior of technology into the analyses.

To test the research hypotheses, a set of variables that allowed the exploration of the use of mobile devices for PAT in more detail was taken into account. One central construct in the analyses was satisfaction with physical fitness. Satisfaction is an individual’s mental state and a subjective measurement of his or her self-evaluated contentment. In the context of physical activity, this means that an individual is at ease with his or her physical activity levels (eg, he or she is satisfied with current fitness levels and the frequency and the intensity of exercise). Similar to previous research [16], an individual’s satisfaction with his or her physical fitness was measured with the question *How satisfied are you currently with your physical fitness?* on 5-point Likert scales (0=not at all satisfied to 4=fully satisfied).

**Figure 1:**
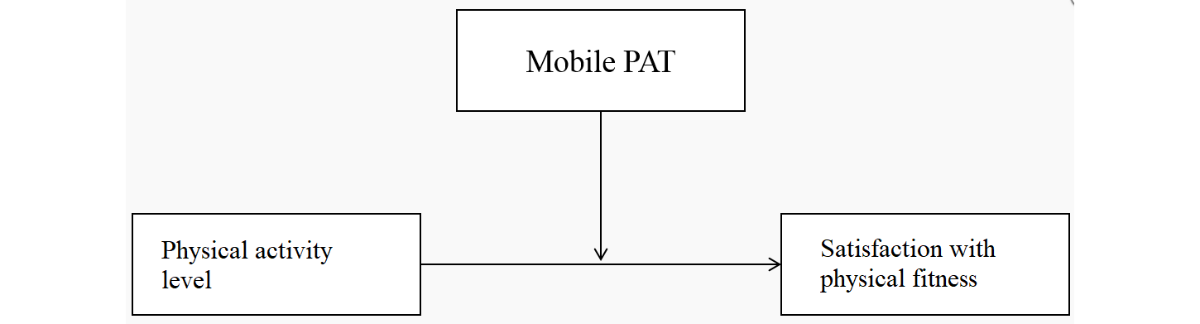
Research model of moderation analysis to show the effect of physical activity level on satisfaction with physical fitness moderated by physical activity tracking (PAT).

The level of physical activity was also measured using a self-reported question, as in previous studies [43]. Participants were asked, *How often do you exercise normally?* and evaluated on a 6-point scale (0=never to 5=daily). Age (*continuous*, in years), gender (*female* or *male*), and income (1≤CHF 4000, 2=between CHF 4000-9000, and 3≥CHF 9000 per month) were included as control variables because previous research has shown that a person’s assessment of satisfaction is affected by these sociodemographic variables [44].

### Statistical Analyses

SPSS version 24 (IBM Statistics) was used for statistical analyses. Missing data were excluded listwise. As a first step, we distinguished between the PAT and noPAT groups. To compare the characteristics of the 2 groups, we calculated *t* tests for independent samples, the chi-square statistics, and Cramér V. Furthermore, we evaluated Cohen *d* to estimate the practical relevance of the differences.

To test the research hypotheses, we performed multiple linear regression analyses (OLS). The dependent variable in these analyses was satisfaction with physical fitness (on a 5-point Likert scale). The independent variables included age (in years, mean centered), gender (reference female), income (reference more than CHF 9000), level of physical activity (on a 6-point scale), and PAT group (reference noPAT). In the first model (main-effect model), the main effects of the independent variables are reported. In a second model (interaction-effect model), we additionally included an interaction term between PAT and physical activity level. The interaction was calculated by multiplying the variables level of physical activity and PAT. Including the interaction term between physical activity level and PAT allowed us to model the effect of physical activity level on satisfaction with physical fitness depending on whether individuals tracked their physical activity with mobile devices or apps. This process is an example of moderation analysis (simple moderation analysis). As described by Hayes [45], a moderation analysis allows researchers “to determine whether a certain variable influences or is related to the size of one variable’s effect on another.” In this study, we expect the effect of physical activity level on satisfaction with physical fitness to depend on PAT, rendering PAT as the moderator variable. The conceptual model of moderation analysis according to Hayes [45] is illustrated in Figure 1.

## Results

### Characteristics of Physical Activity Tracking and No Physical Activity Tracking Groups

Altogether, 18.9% (136/719) participants used a device to track their physical activity and were therefore considered as members of the PAT group. Participants in the PAT group used an activity tracker (59.6%, 81/136), smartwatch (12.5%, 17/136), and/or smartphone or tablet app (57.4%, 78/136) to track their physical activity. Most members of the PAT group (74.3%, 101/136) used a single device to track their physical activity, whereas only a minority used 2 (22.0%, 30/136) or 3 (3.7%, 5/136) devices.

The remaining 81.1% (583/719) of participants used at least 1 mobile device but for purposes other than tracking their physical activity, and they were considered as members of the noPAT group.

On comparing participants in the PAT and noPAT groups (see Table 1), it was observed that there was no significant difference in age (*t*_717_=0.96, *P*=.34, Cohen *d*=0.010). The mean age of the members of the PAT group was 61.95 (SD 9.43) years. Participants in this group ranged in age from 50 to 86 years. The members of the noPAT group had a mean age of 62.81 (SD 9.43) years and ranged in age from 50 to 88 years. Group membership differed significantly according to gender (χ^2^_1_=5.8, *P*=.02); 58.8% (80/136) of the individuals in the PAT group were male, whereas males comprised 47.3% (276/583) of the noPAT group. There was no significant relationship between group membership and income (*V*=.05, *P*=.47). In both groups, the majority of individuals had an income between CHF 4000 and 9000. Specifically, 50.4% (60/119) of the individuals in the PAT group fell into this income category, and 56.5% (277/490) of the individuals in the noPAT group fell into this same category as well. No significant difference was observed in members’ satisfaction with physical fitness for the 2 groups (*t*_716_=1.00; *P*=.32; Cohen *d*=0.187); this was also true for the frequency of physical activity (*t*_245_=1.27; *P*=.21; Cohen *d*=0.112). Within the PAT group, there was no significant correlation between the number of devices that were used for mobile PAT and satisfaction with physical fitness (*r*=.07; *P*=.45).

**Table 1:** Characteristics of the physical activity tracking and no physical activity tracking groups (only respondents who own a mobile device, n=719).

Characteristics		User group		Significance	*P* value	Cohen *d*
		PAT^a^	noPAT			
Age (years), mean (SD)		61.95 (9.43)	62.81 (9.43)	*t*_717_=0.96	0.34	0.01
**Gender, n (%)**						
	Men	80 (58.8)	276 (47.3)	χ^2^_1_=5.8	0.02	—^b^
	Women	56 (41.2)	307 (52.7)	χ^2^_1_=5.8	0.02	—
**Income, n (%)**						
	<CHF 4000	17 (14.3)	65 (13.3)	*V*=.05	0.47	—
	Between CHF 4000-9000	60 (50.4)	277 (56.5)	*V*=.05	0.47	—
	>CHF 9000	42 (35.3)	148 (30.2)	*V*=.05	0.47	—
Satisfaction with physical fitness^c^, mean (SD)		2.81 (1.00)	2.90 (0.92)	*t*_716_=1.00	0.32	0.187
Mean frequency of physical activity^d^, mean (SD)		3.72 (1.13)	3.58 (1.42)	*t*_245_=1.27	0.21	0.112

^a^PAT: physical activity tracking.

^b^Not applicable.

^c^Measured on a 5-point Likert scale.

^d^Measured on a 6-point scale.

### Predictors of Satisfaction With Physical Fitness

To analyze the effects of physical activity levels and PAT on satisfaction with physical fitness, a multiple linear regression (based on a simple moderation analysis) was performed. The level of physical activity, PAT, and interaction term between both variables were included as independent variables in the full model (model 2). The findings were compared with a model including only the main effects (model 1). In addition, we considered age, gender, and income as control variables in both regression models. All persons who used a mobile device in general were included in the analysis.

The main-effect model (model 1) has an adjusted *R*^*2*^ of .08 and explains a significant amount of variance in terms of satisfaction with physical fitness (*F*_6,601_=10.39, *P*<.001). Frequent physical activity was a significant predictor (*P*<.001), whereas inclusion in the PAT group showed no significance (*P*=.46). Likewise, age, gender, and income were not significant predictors (see Table 2).

Overall, the interaction-effect model (model 2) demonstrates a significant amount of variance within people’s satisfaction with physical fitness (*F*_7,600_=9.65, *P*<.001), with an adjusted *R*^2^ of .09. The model revealed that frequent physical activity (*P*<.001) and inclusion in the PAT group (*P*=.02) were significant predictors of satisfaction with physical fitness. The interaction term given by *PAT* * *physical activity* also showed significance (*P*=.03). Again, age, gender, and income were no significant predictors for individual satisfaction with physical fitness (see Table 2).

**Figure 2:**
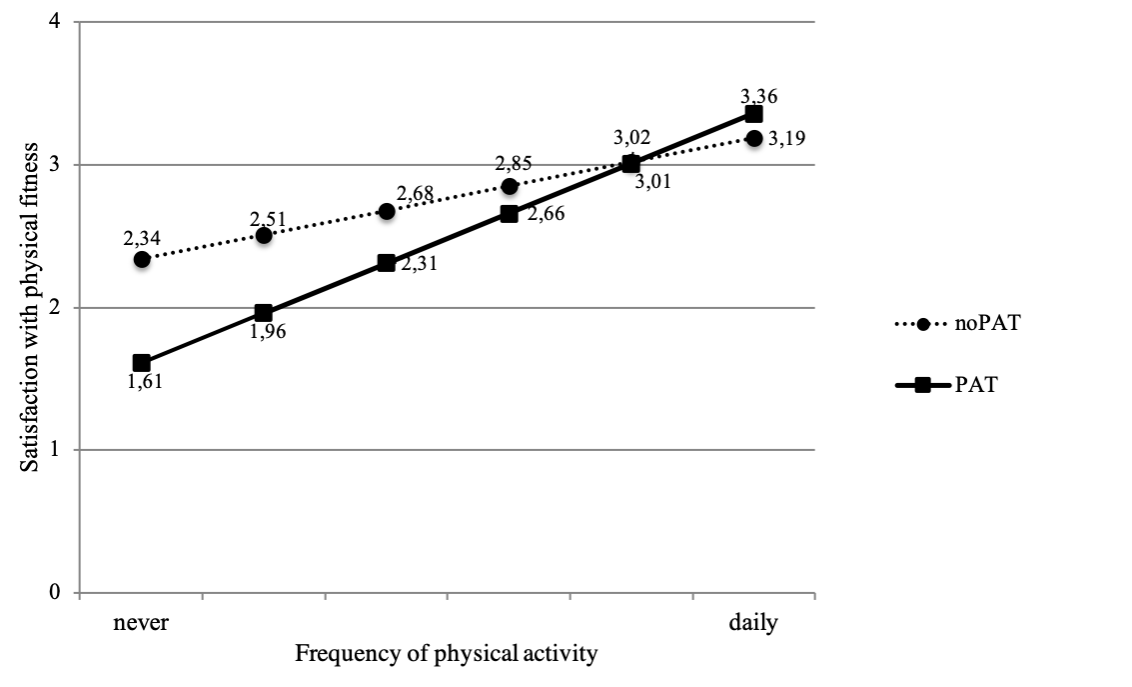
Interaction diagram of the effects of physical activity level and physical activity tracking (PAT) on satisfaction with physical fitness. Graphic representation of unstandardized regression estimates for different groups depending on physical activity level and PAT. Values displayed for a female with mean age and medium income (between CHF 4000-9000). Frequency of physical activity was measured using a 6-point scale from never to daily and satisfaction with physical fitness was measured using a 5-point Likert scale.

In both models, higher levels of physical activity had a positive effect on satisfaction with physical fitness (main-effect model: beta=.27, *P*<.001; interaction-effect model: beta=.24, *P*<.001). People who were more physically active were more satisfied with their physical fitness status. Only within the interaction-effect model was there a significant effect of PAT group (beta=−.31, *P*=.02), indicating a significant difference in satisfaction with physical fitness between members of the PAT group and the noPAT group, when individuals are *never* physically active. Furthermore, within this model, the interaction term *PAT* * *physical activity* modeled the conditional effect of physical activity level on satisfaction with physical fitness depending on group membership (PAT or noPAT). The positive interaction effect (beta=.30, *P*=.03) indicated that the effect of physical activity on satisfaction was stronger for people in the PAT group, meaning that, when using mobile devices for PAT, the positive effect of physical activity level on satisfaction with physical fitness was stronger (see Figure 2). As shown in Figure 2, no differences in the level of satisfaction with physical fitness were observed between members of the PAT and the noPAT group when individuals were physically active on a daily basis.

**Table 2:** Multiple linear regression analysis of the predictors of satisfaction (measured using a 5-point Likert scale) with physical fitness (simple moderation analysis on only respondents who own a mobile device, n=719).

Predictor	Model 1: main-effect model^a^			Model 2: interaction-effect model^b^		
	b (SE)	Beta	*P* value	b (SE)	Beta	*P* value
Constant	2.18 (0.14)	—^c^	<.001	2.26 (0.14)	—	<.001
Age^d^	0.01 (0)	.06	.12	0.01 (0)	.06	.12
Gender: male (reference female)	0.01 (0.07)	.01	.84	0.01 (0.07)	.01	.89
Income: <CHF 4000 (reference >CHF 9000)	−0.22 (0.12)	−.08	.07	−0.22 (0.12)	−.08	.07
Income: between CHF 4000-9000 (reference >CHF 9000)	0.08 (0.08)	.04	.33	0.08 (0.08)	.04	.34
Physical activity^e^	0.19 (0.03)	.27	<.001	0.17 (0.03)	.24	<.001
PAT^f^ group (reference noPAT)	−0.07 (0.09)	−.03	.46	−0.73 (0.31)	−.31	.02
Interaction: PAT * physical activity	—	—	—	0.18 (0.08)	.30	.03

^a^Adjusted *R*^2^=.08, *F*_6,601_=10.39, *P*<.001.

^b^Adjusted *R*^2^=.09, *F*_7,600_=9.65, *P*<.001.

^c^Not applicable.

^d^Mean centered.

^e^Measured on a 6-point scale (*never* to *daily*).

^f^PAT: physical activity tracking.

## Discussion

### Principal Findings

This study was the first conducted in Switzerland—and to our best knowledge, among the first internationally—to examine the relevance of PAT in the relationship between physical activity level and satisfaction with physical fitness in a representative sample of individuals aged 50 years and older. Our analyses were based on 719 older individuals who use mobile devices in their everyday life.

Bivariate results showed that people who tracked their physical activity using a mobile device were more likely to be male. In the multivariate analysis, we addressed the relationship between physical activity level and satisfaction with physical fitness and mobile PAT in more detail. Examining the relevance of PAT for the positive effects of physical activity on satisfaction with physical fitness is an important research topic, as this might contribute to the well-being of older persons.

The multiple regression models revealed that physical activity level was a positive and significant predictor of satisfaction with physical fitness in both the main-effect model and the interaction-effect model. Individuals who were physically active more often were more satisfied with their physical fitness. These findings are in line with our research hypothesis (H1). Furthermore, we tested whether this relationship was influenced by the use of mobile devices for PAT. We found a significant positive interaction effect between physical activity level and PAT on satisfaction with physical activity; the positive effect of physical activity levels on satisfaction was stronger for people using PAT (H2). Results also showed that PAT had no positive effects on satisfaction with physical fitness in the main-effect model and for individuals who were not physically active on a regular basis (see Figure 2). These findings need to be discussed on several levels.

### Comparison With Previous Work

In general, people who track their physical activity with a mobile device are more aware of how active they actually are, as compared with individuals who do not track their physical activity [32]. Research showed that they can better understand correlations between their behavior and possible health outcomes [46]. Therefore, when they indulge in frequent physical activity, it has a more positive effect on their level of satisfaction. This relationship was also identified in this study: PAT could add to the positive effects of physical activity on satisfaction with physical fitness. In contrast, it is reasonable to assume that individuals who are less physically active and use mobile devices for PAT do not overestimate their activity level, as often happens in subjective measurements of physical activity [47], especially for older persons [48]. As they are likely more aware of not meeting physical activity guidelines—such as the World Health Organization’s goal of reaching 10,000 steps per day [3]—they are consequently less satisfied with their physical fitness. Moreover, our results support this assumption as shown by the negative effect of PAT on satisfaction with physical fitness for individuals who were not physically active.

This negative effect of PAT on satisfaction with physical fitness is an important finding in the evaluation of possible impacts of PAT on well-being and motivation for physical activity. The positive effects of PAT cannot be assumed for everyone. Our results show that in the case of less frequent (not daily) physical activity, PAT might have negative consequences for the users. On the other hand, the use of PAT supports satisfaction with physical fitness for older adults who are physically active on a daily basis. This is an interesting result because this relationship among physical activity, PAT, and satisfaction might also influence the long-term use of PAT and the motivation for physical activity. Further research is needed to investigate these findings in more detail.

To analyze these mechanisms in greater detail, future studies should try to objectively measure the frequency and level of physical activity with tracking technology and electronic momentary assessments to get a more reliable measurement. A further point of interest might be whether the results would differ if activity had been measured using time instead of frequency. Some older people may be active frequently but have a short overall duration, and others may be active infrequently with a long weekly duration.

Furthermore, it can be assumed that activity trackers, or other devices and apps for PAT, are often purchased because individuals are unsatisfied with their current situation, such as their fitness level. In this case, there might be a reverse causality: negative satisfaction has an effect on using PAT and not vice versa. A recent study [43] provides some arguments for this assumption and shows that the health apps people have installed on their smartphone do not represent their actual behaviors but rather the behaviors they would like to change. In this way, PAT might be used for self-optimization. This will not happen in the short term, and positive effects might only be observable after longer periods of use. This study does not consider the duration of use. However, previous studies have shown that activity trackers are often not used for longer than half a year [49]. Individuals probably stop using devices for tracking physical activity before positive effects can be observed. To understand this issue, it is also important to know why individuals originally get the devices. Different motivations and intended uses can be assumed depending on whether the device is bought as a lifestyle device or prescribed by a physician and integrated into programs offered by health insurance providers.

On the basis of the empirical data in this study, it is not possible to reach a concrete conclusion, and the above assumptions need to be examined in greater detail. Future work could extend this research by using longitudinal data. This would allow for the examination of intraindividual change processes and objective tracking data, as it would be possible to study the relevant motivational mechanisms and relationships over time.

### Practical Implications

With regard to the devices used for PAT, it should be noted that most current devices and apps have been developed without considering health psychology or gerontology theories. It is therefore unlikely that the devices used for PAT have been customized for long-term use or sustainable success among older users. A qualitative study with activity trackers showed that older adults might feel overstrained by predetermined goals that are not individualized [12]. The general guidelines for physical activity might exceed older adults’ abilities, resulting in certain injuries or health problems. Therefore, defining appropriate goals is difficult for the group of older people. One recommendation could be to use official institutional guideline values as a starting reference point (eg, [3,4]) and then adapt these to the individual. Such an adaptation should be based on subjectively and objectively measured user performance, with the goal of avoiding feelings of being overstrained. A review of free coaching apps even showed that almost none of these apps were evidence-based or suitable for beginners [50]. Structured physical activity interventions with systematic training progression are important for older adults who want to lead healthier lives [51]. Therefore, scientists should assume responsibility for integrating evidence-based theories into the development of new technologies and mobile apps. One possibility might be to add dynamic concepts that allow for customization for different users as well as for individual development over time. In addition, physical activity–tracking products (ie, activity trackers as well as smartwatches, smartphones, and tablets) must target the specific needs, especially in terms of usability and usefulness of older users [52,53].

### Limitations

As this study had a specific regional focus, the generalization of our findings is limited. The data provide only a cross-sectional view of the phenomena, but it is likely that there will be a further increase in mobile activity and health tracking among older individuals in general. Further research, possibly including longitudinal data, is required to examine the potential increase and to make inferences related to mobile activity tracking and subjective well-being and health over time and across individuals. Furthermore, it is possible that participants understand questions on subjective measurements (eg, the frequency of physical activity and their satisfaction with physical fitness) differently, which can affect the results. Furthermore, we did not have any information on the intensity of using PAT. We could only distinguish the PAT and noPAT groups. However, a more active use of PAT in everyday life might have greater effects. A qualitative study showed that older people often passively use devices for PAT in their everyday lives [21]. The effects of more irregular use of PAT (eg, tracking less frequent hiking tours) also could not be analyzed within this study, as the regular use of PAT was the focus of analysis. In future research, these aspects need to be clarified; they could also be the focus of future quantitative studies.

We only studied satisfaction with physical fitness. This key outcome of the study was only measured using a single item, which might be subject to bias. It should be noted that an overall evaluation of physical activity and its effects on subjective experiences should ideally be realized in a more differentiated matter. A 1-item measure is problematic, as the different dimensions of the phenomenon cannot be distinguished. However, the available variables used in our secondary data enabled us to analyze the relevance of mobile PAT in terms of the relationship between physical activity and subjective fitness in an exploratory way. Future studies should therefore operationalize this concept through a multidimensional approach and extend this view by both including other measures of satisfaction and quality of life and by using validated questionnaires to confirm the findings.

In addition, in this first study, data on important background factors (eg, technology knowledge, attitudes toward technology, and objective health status), fitness status (eg, objective measures of exercise, fitness status, and activity levels), everyday life factors (eg, coping with activities of daily life and social contact), and psychological factors (eg, attitudes toward health prevention and one’s own life and aging, personality, and well-being) were unavailable. Further studies with a wider range of variables and a longitudinal design are therefore required to examine the study topic in greater detail.

### Conclusions

This study provides some evidence that PAT can enhance the positive effect of physical activity levels on satisfaction with physical fitness. The results indicate the potential of mobile PAT to improve the well-being of older adults. Especially for older individuals, mobile devices can allow for the easy longitudinal monitoring and documentation of their health status. However, the results also raise new issues concerning the relationship between PAT and satisfaction with physical fitness. PAT showed a negative effect on satisfaction with physical fitness for individuals who were not physically active on a regular basis. We discussed this finding in the context of self-optimization through PAT, long-term use of the devices, and older adults’ specific requirements in terms of usability and usefulness. Further research is required in this fast-moving field to understand relevant processes and causalities in greater detail.

## Abbreviations

noPAT group: no physical activity tracking group
OLS: ordinary least squares
PAT: physical activity tracking
PAT group: physical activity tracking group

## References

[1] Lee IM, Shiroma EJ, Lobelo F, Puska P, Blair SN, Katzmarzyk PT, Lancet Physical Activity Series Working Group (2012). Effect of physical inactivity on major non-communicable diseases worldwide: an analysis of burden of disease and life expectancy. Lancet.

[2] Hallal PC, Andersen LB, Bull FC, Guthold R, Haskell W, Ekelund U, Lancet Physical Activity Series Working Group (2012). Global physical activity levels: surveillance progress, pitfalls, and prospects. Lancet.

[3] World Health Organization.

[4] US Department of Health and Human Services (2008). Office of Disease Prevention and Health Promotion.

[5] Haskell WL, Blair SN, Hill JO (2009). Physical activity: health outcomes and importance for public health policy. Prev Med.

[6] Reiner M, Niermann C, Jekauc D, Woll A (2013). Long-term health benefits of physical activity--a systematic review of longitudinal studies. BMC Public Health.

[7] Peel NM, McClure RJ, Bartlett HP (2005). Behavioral determinants of healthy aging. Am J Prev Med.

[8] Bherer L, Erickson KI, Liu-Ambrose T (2013). A review of the effects of physical activity and exercise on cognitive and brain functions in older adults. J Aging Res.

[9] Rasciute S, Downward P (2010). Health or happiness? What is the impact of physical activity on the individual?. Kyklos.

[10] Phillips AC, Der G, Carroll D (2010). Self-reported health, self-reported fitness, and all-cause mortality: prospective cohort study. Br J Health Psychol.

[11] Burton NW, Walsh A, Brown WJ (2008). It just doesn't speak to me: mid-aged men's reactions to '10,000 Steps a Day'. Health Promot J Austr.

[12] Schlomann A, von Storch K, Rasche P, Rietz C (2016). Means of motivation or of stress? The use of fitness trackers for self-monitoring by older adults. HeilberufeScience.

[13] Bize R, Johnson JA, Plotnikoff RC (2007). Physical activity level and health-related quality of life in the general adult population: a systematic review. Prev Med.

[14] Ruseski JE, Humphreys BR, Hallman K, Wicker P, Breuer C (2014). Sport participation and subjective well-being: instrumental variable results from German survey data. J Phys Act Health.

[15] Garatachea N, Molinero O, Martínez-García R, Jiménez-Jiménez R, González-Gallego J, Márquez S (2009). Feelings of well being in elderly people: relationship to physical activity and physical function. Arch Gerontol Geriatr.

[16] Wicker P, Frick B (2015). The relationship between intensity and duration of physical activity and subjective well-being. Eur J Public Health.

[17] Pawlowski T, Downward P, Rasciute S (2011). Subjective well-being in European countries—on the age-specific impact of physical activity. Eur Rev Aging Phys Act.

[18] Wicker P, Coates D, Breuer C (2015). The effect of a four-week fitness program on satisfaction with health and life. Int J Public Health.

[19] Lera-López F, Ollo-López A, Sánchez-Santos JM (2016). How does physical activity make you feel better? The mediational role of perceived health. Appl Res Qual Life.

[20] Ehlers D, Salerno EA, Aguiñaga S, McAuley E (2018). Noba Scholar.

[21] Schlomann A (2017). A case study on older adults’ long-term use of an activity tracker. Gerontechnology.

[22] Lamonaca F, Polimeni G, Barbé K, Grimaldi D (2015). Health parameters monitoring by smartphone for quality of life improvement. Measurement.

[23] Swan M (2012). Sensor mania! The internet of things, wearable computing, objective metrics, and the quantified self 2.0. J Sens Actuator Netw.

[24] Vashist SK, Schneider EM, Luong JH (2014). Commercial smartphone-based devices and smart applications for personalized healthcare monitoring and management. Diagnostics (Basel).

[25] Monroe CM, Thompson DL, Bassett DR, Fitzhugh EC, Raynor HA (2015). Usability of mobile phones in physical activity–related research: a systematic review. Am J Health Educ.

[26] Poirier J, Bennett WL, Jerome GJ, Shah NG, Lazo M, Yeh H, Clark JM, Cobb NK (2016). Effectiveness of an activity tracker- and internet-based adaptive walking program for adults: a randomized controlled trial. J Med Internet Res.

[27] Steinert A, Wegel S, Steinhagen-Thiessen E (2015). Self-monitoring of physical activity by the elderly. HeilberufeScience.

[28] Cugelman B (2013). Gamification: what it is and why it matters to digital health behavior change developers. JMIR Serious Games.

[29] Fritz T, Huang E, Murphy G, Zimmermann T (2014). Persuasive technology in the real world: a study of long-term use of activity sensing devices for fitness. Proceedings of the SIGCHI Conference on Human Factors in Computing Systems.

[30] Lister C, West JH, Cannon B, Sax T, Brodegard D (2014). Just a fad? Gamification in health and fitness apps. JMIR Serious Games.

[31] Appelboom G, Camacho E, Abraham ME, Bruce SS, Dumont EL, Zacharia BE, D'Amico R, Slomian J, Reginster JY, Bruyère O, Connolly Jr ES (2014). Smart wearable body sensors for patient self-assessment and monitoring. Arch Public Health.

[32] Morgan H (2016). 'Pushed' self-tracking using digital technologies for chronic health condition management: a critical interpretive synthesis. Digit Health.

[33] Direito A, Jiang Y, Whittaker R, Maddison R (2015). Apps for IMproving FITness and increasing physical activity among young people: the AIMFIT pragmatic randomized controlled trial. J Med Internet Res.

[34] Safran NJ, Madar Z, Shahar DR (2015). The impact of a web-based app (eBalance) in promoting healthy lifestyles: randomized controlled trial. J Med Internet Res.

[35] Walsh JC, Corbett T, Hogan M, Duggan J, McNamara A (2016). An mHealth intervention using a smartphone app to increase walking behavior in young adults: a pilot study. JMIR Mhealth Uhealth.

[36] Dallinga JM, Mennes M, Alpay L, Bijwaard H, de la Faille-Deutekom MB (2015). App use, physical activity and healthy lifestyle: a cross sectional study. BMC Public Health.

[37] Drewnowski A, Monsen E, Birkett D, Gunther S, Vendeland S, Su J, Marshall G (2003). Health screening and health promotion programs for the elderly. Dis Manag Health Out.

[38] Ehn M, Eriksson LC, Åkerberg N, Johansson A (2018). Activity monitors as support for older persons' physical activity in daily life: qualitative study of the users' experiences. JMIR Mhealth Uhealth.

[39] Kim BY, Lee J (2017). Smart devices for older adults managing chronic disease: a scoping review. JMIR Mhealth Uhealth.

[40] Broekhuizen K, de Gelder J, Wijsman CA, Wijsman LW, Westendorp RG, Verhagen E, Slagboom PE, de Craen AJ, van Mechelen W, van Heemst D, van der Ouderaa F, Mooijaart SP (2016). An internet-based physical activity intervention to improve quality of life of inactive older adults: a randomized controlled trial. J Med Internet Res.

[41] Seifert A, Schlomann A, Rietz C, Schelling HR (2017). The use of mobile devices for physical activity tracking in older adults' everyday life. Digit Health.

[42] Universität Zürich.

[43] Ernsting C, Dombrowski SU, Oedekoven M, O Sullivan JL, Kanzler M, Kuhlmey A, Gellert P (2017). Using smartphones and health apps to change and manage health behaviors: a population-based survey. J Med Internet Res.

[44] Daig I, Herschbach P, Lehmann A, Knoll N, Decker O (2009). Gender and age differences in domain-specific life satisfaction and the impact of depressive and anxiety symptoms: a general population survey from Germany. Qual Life Res.

[45] Hayes AF (2013). Introduction to Mediation, Moderation, and Conditional Process Analysis: A Regression-Based Approach (Methodology in the Social Sciences).

[46] Bhavnani SP, Narula J, Sengupta PP (2016). Mobile technology and the digitization of healthcare. Eur Heart J.

[47] Shephard RJ (2003). Limits to the measurement of habitual physical activity by questionnaires. Br J Sports Med.

[48] Tudor-Locke C, Craig CL, Aoyagi Y, Bell RC, Croteau KA, de Bourdeaudhuij I, Ewald B, Gardner AW, Hatano Y, Lutes LD, Matsudo SM, Ramirez-Marrero FA, Rogers LQ, Rowe DA, Schmidt MD, Tully MA, Blair SN (2011). How many steps/day are enough? For older adults and special populations. Int J Behav Nutr Phys Act.

[49] Ledger D, McCaffrey D Endevour Partners.

[50] Modave F, Bian J, Leavitt T, Bromwell J, Harris IC, Vincent H (2015). Low quality of free coaching apps with respect to the American College of Sports Medicine Guidelines: a review of current mobile apps. JMIR Mhealth Uhealth.

[51] Pelssers J, Delecluse C, Opdenacker J, Kennis E, van Roie E, Boen F (2013). “Every Step Counts!”: effects of a structured walking intervention in a community-based senior organization. J Aging Phys Act.

[52] Preusse KC, Mitzner TL, Fausset CB, Rogers WA (2017). Older adults' acceptance of activity trackers. J Appl Gerontol.

[53] Seifert A, Schelling H (2015). Mobile use of the internet using smartphones or tablets by Swiss people over 65 years. Gerontechnology.

